# How AI disclosure influences consumers' purchase intention toward AI-generated Chinese ethnic-minority fashion: the roles of perceived usefulness, perceived authenticity, and algorithm aversion

**DOI:** 10.3389/fpsyg.2026.1905449

**Published:** 2026-10-21

**Authors:** Kuilin Wei, Jun Liu, Shuai Wang, Yue Sun

**Affiliations:** 1 College of Art, Cheongju University, Cheongju, Republic of Korea; 2 School of Design, Fujian University of Technology, Fuzhou, China

**Keywords:** AI disclosure, AI-generated ethnic fashion, algorithm aversion, perceived authenticity, perceived usefulness

## Abstract

**Background:**

With the growing application of generative artificial intelligence (AI) in fashion design and cultural and creative industries, AI-generated Chinese ethnic-minority fashion has created new opportunities for the contemporary transformation of traditional culture. However, whether consumers are willing to accept such products and how AI disclosure influences their evaluations and purchase intentions remain unclear. Drawing on the Stimulus–Organism–Response (S–O–R) model, this study examines the mechanisms through which AI disclosure, design aesthetics, cultural identity, perceived usefulness, perceived authenticity, and algorithm aversion influence consumers' purchase intentions toward AI-generated Chinese ethnic-minority fashion.

**Methods:**

A questionnaire survey combined with a scenario-based experiment was employed. Six AI-generated fashion illustrations derived from Tujia costume elements were developed and selected as stimulus materials. Participants were randomly assigned to either an AI disclosure group or a non-disclosure group. Data were collected through Tencent Questionnaire, yielding 410 valid responses. Partial Least Squares Structural Equation Modeling (PLS-SEM) and Importance–Performance Map Analysis (IPMA) were employed to examine the structural relationships, mediating effects, moderating effects, and practical priorities for improvement.

**Results:**

Design aesthetics and cultural identity influenced purchase intention through perceived usefulness and perceived authenticity, with perceived usefulness emerging as the strongest predictor of purchase intention. AI disclosure significantly reduced design aesthetics, cultural identity, perceived usefulness, and perceived authenticity. Algorithm aversion did not significantly moderate the relationship between perceived usefulness and purchase intention but significantly strengthened the positive effect of perceived authenticity on purchase intention.

**Conclusions:**

This study reveals the multiple psychological pathways through which AI disclosure shapes consumers' evaluations of AI-generated ethnic cultural fashion. The findings further indicate that algorithm aversion alters the extent to which consumers rely on authenticity judgments when forming purchase intentions. This study extends the application of the Stimulus–Organism–Response (S–O–R) model to the context of AI-generated cultural products and provides practical implications for balancing AI transparency, cultural authenticity, and the practical value of products.

## 1 Introduction

Tujia costume is an important component of China's ethnic-minority cultural heritage, embodying the historical memory, aesthetic values, and cultural symbols of the Tujia people (He, 2021). Its clothing elements not only reflect the distinctive artistic expression of Tujia culture but also demonstrate the continuity and transmission of ethnic culture through visual symbols (Yuan and Somthai, 2025). As lifestyles and consumer demands continue to evolve, traditional ethnic costumes face a range of challenges, including weakened cultural transmission, insufficient design transformation, and limited market acceptance (Fu and Liang, 2022). In recent years, digital technologies have created new opportunities for integrating cultural heritage, traditional craftsmanship, and creative industries, enabling traditional cultural elements to enter contemporary consumption contexts through digital expression and design innovation (Jiang and Fu, 2017; Li, 2020). At the same time, generative AI has gradually emerged as an important technological tool for creative production (Biliakovych et al., 2025; Lyu et al., 2024). Against this backdrop, AI-generated Chinese ethnic-minority fashion represents not only a new form of design but also a new research context for exploring the design transformation and market acceptance of ethnic fashion. Given that Tujia costume possesses distinctive ethnic cultural symbols, a well-established clothing system, and a solid foundation of academic research (Yuan and Somthai, 2025), this study selects Tujia costume as a representative case of Chinese ethnic-minority fashion for empirical investigation.

Previous studies have shown that consumers do not necessarily accept products labeled as AI-designed (Lee and Kim, 2024). When encountering such designs, consumers evaluate whether the products convey aesthetic, functional, and symbolic meanings through their assessment of design aesthetics (Li and Li, 2022). Research has also indicated that the incorporation of traditional cultural symbols can enhance consumers' cultural identity, enabling products to serve as vehicles for cultural expression and identity representation (Liu and Zhao, 2024; Zong et al., 2023). Furthermore, consumers evaluate whether a design possesses practical value, referred to as perceived usefulness (Ruiz-Viñals et al., 2024), and whether it authentically and appropriately represents relevant cultural knowledge, referred to as perceived authenticity (Kreuzbauer and Keller, 2017). In the context of AI-generated design, consumers generally prefer human-designed apparel over AI-designed apparel (Lee and Kim, 2024). Consequently, even when AI-generated designs are perceived as highly useful or authentic, a high level of algorithm aversion may still weaken the influence of these positive psychological evaluations on purchase intention (Mahmud et al., 2022). Therefore, consumers' acceptance of AI-generated Chinese ethnic-minority fashion may not be determined solely by visual evaluations or attitudes toward technology. Instead, it is likely shaped by multiple psychological evaluations, including design aesthetics, cultural identity, perceived usefulness, and perceived authenticity.

Although previous studies have separately examined consumer responses to AI disclosure, aesthetic perceptions of AI-generated designs, cultural identity and authenticity evaluations elicited by traditional cultural elements, the role of perceived usefulness in consumer decision-making, and the influence of algorithm aversion on consumer behavior (Bellaiche et al., 2023; Fu and Liang, 2022; Lee and Kim, 2024; Prashar et al., 2017; Zhou et al., 2022), few studies have adopted an integrated perspective that incorporates AI disclosure, design aesthetics, cultural identity, perceived usefulness, perceived authenticity, and algorithm aversion within a single theoretical framework. As a result, the relationships and underlying mechanisms through which these factors jointly shape purchase intention remain insufficiently understood. To address this gap, the present study develops a Stimulus–Organism–Response (S–O–R) model in which AI disclosure serves as the external stimulus (S), design aesthetics, cultural identity, perceived usefulness, and perceived authenticity represent internal psychological states (O), and purchase intention is treated as the behavioral response (R). The proposed model is examined using Partial Least Squares Structural Equation Modeling (PLS-SEM) and Importance–Performance Map Analysis (IPMA) (Hair et al., 2019; Riou et al., 2016).

The theoretical contribution of this study lies in extending the S–O–R model to the consumption context of AI-generated Tujia fashion and uncovering the psychological mechanisms through which AI disclosure, design aesthetics, cultural identity, perceived usefulness, and perceived authenticity jointly influence purchase intention. In addition, the study further clarifies the role of algorithm aversion in shaping the relationship between consumers' psychological evaluations and their purchase intentions. From a practical perspective, the findings provide consumer behavior insights for AI-assisted ethnic fashion design, the contemporary transformation of Tujia cultural elements, and the development of related cultural and creative products.

## 2 Literature review and research hypotheses

### 2.1 Stimulus–Organism–Response (S–O–R) model

The Stimulus–Organism–Response (S–O–R) model was originally proposed by Mehrabian and Russell (1974). Rooted in environmental psychology, the model has been widely applied in consumer behavior and information systems research (Jacoby, 2002). According to the S–O–R framework, external environmental stimuli (S) influence individuals' internal psychological states (O), which subsequently lead to corresponding behavioral responses (R) (Jacoby, 2002; Zhou et al., 2022).

In consumer behavior research, the S–O–R model explains how consumers develop internal psychological evaluations in response to product characteristics, information presentation, and environmental stimuli, which in turn shape behavioral responses such as purchase intention (Jacoby, 2002; Prashar et al., 2017; Zhou et al., 2022). The model has been extensively used to explain consumer decision-making processes involving emerging technology products, digital content products, cultural and creative products, and fashion products (Jacoby, 2002; Nam et al., 2021; Zhou et al., 2022). Therefore, the S–O–R model provides a suitable theoretical framework for examining the mechanisms through which AI-generated Chinese ethnic-minority fashion influences consumers' purchase intentions.

This study adopts the S–O–R model as its fundamental analytical framework (Figure 1). AI-generated Chinese ethnic-minority fashion designs and their associated AI disclosure are conceptualized as external stimuli (S). The organism component (O), representing consumers' internal psychological states, is structured into two levels. The first level consists of design aesthetics and cultural identity, reflecting consumers' initial evaluations of the visual quality and cultural significance of the designs. The second level includes perceived usefulness and perceived authenticity, representing consumers' further assessments of the practical value and cultural credibility of the designs. Purchase intention is regarded as the final behavioral response (R). Based on the theoretical logic of the S–O–R model, this study posits that external stimuli influence behavioral responses primarily through the formation and transformation of consumers' internal psychological states. Accordingly, design aesthetics and cultural identity are conceptualized as key antecedents in the process of psychological evaluation. Rather than exerting direct effects on purchase intention, they influence purchase intention indirectly through perceived usefulness and perceived authenticity.

**Figure 1:**
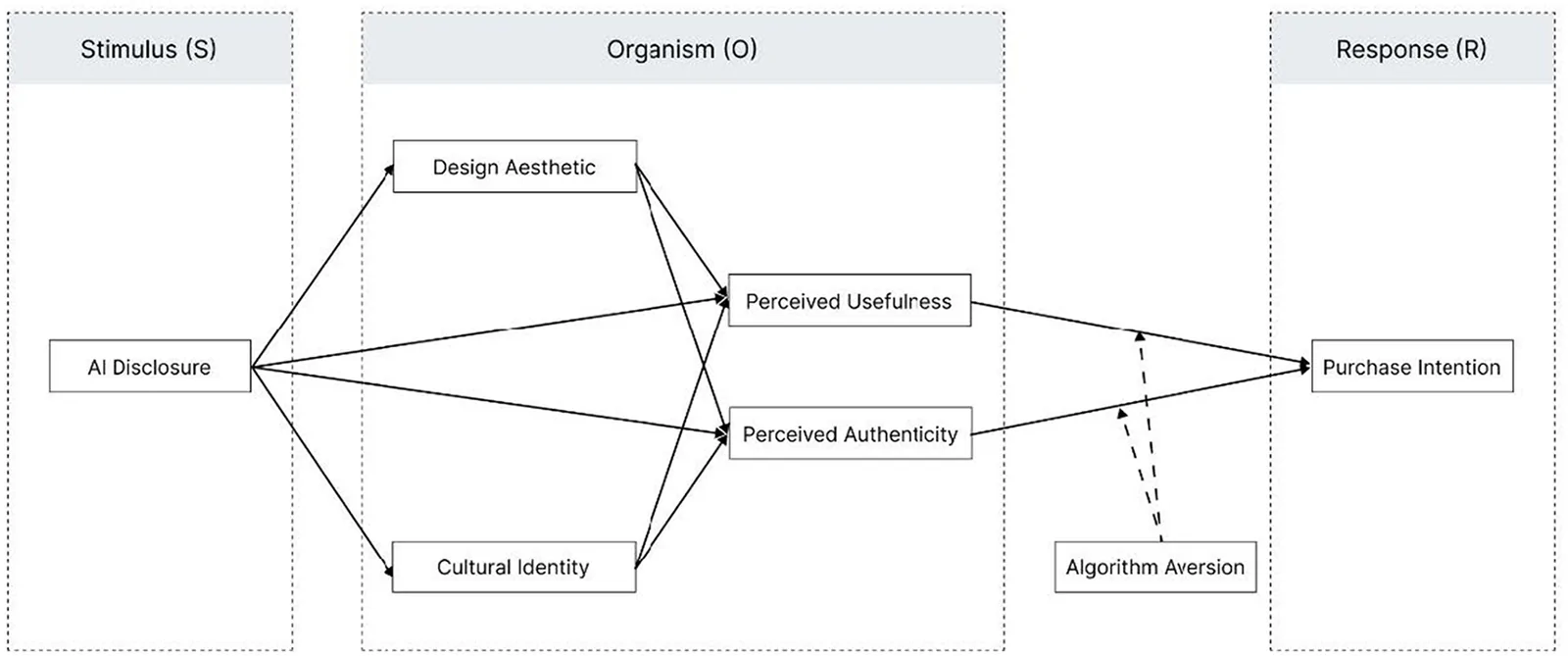
Research model.

In addition, algorithm aversion is incorporated into the model to examine whether it alters the strength of the relationships between perceived usefulness, perceived authenticity, and purchase intention. Based on this theoretical framework, the present study aims to systematically explain how AI disclosure influences consumers' internal psychological evaluations of AI-generated Chinese ethnic-minority fashion and to further reveal how these psychological evaluations are translated into purchase intention.

### 2.2 Research hypotheses

To avoid unnecessarily revalidating well-established relationships, this study distinguishes foundational mechanisms from core theoretical mechanisms. The relationships among design aesthetics, cultural identity, perceived usefulness, perceived authenticity, and purchase intention are treated as the foundational mechanisms underlying the formation and transformation of consumers' evaluations within the Stimulus–Organism–Response (S–O–R) framework. Building upon this foundation, the theoretical analysis focuses on the negative effect of AI disclosure and the boundary role of algorithm aversion, thereby highlighting the mechanisms that offer greater theoretical novelty in the context of AI-generated ethnic cultural fashion.

#### 2.2.1 Design aesthetics

The concept of design aesthetics originates from the broader field of aesthetics (Liu, 2003). As aesthetic research has evolved, increasing scholarly attention has been directed toward design aesthetics (Li and Li, 2022). Previous studies have also identified design aesthetics as an important attribute that reflects both artistic and emotional appeal, defining it as individuals' perceptions of beauty, order, and harmony (Toufani et al., 2017). In the present study, design aesthetics refers to consumers' perceptions and evaluations of the visual presentation and overall aesthetic style of AI-generated Tujia fashion.

Previous research has demonstrated that design aesthetics significantly influences consumers' perceived usefulness (Cyr et al., 2006; Li and Yeh, 2010). In the context of apparel coordination, aesthetic factors have also been found to affect consumers' perceptions of the usefulness of intelligent virtual wardrobes (Perry, 2016). Based on these findings, this study argues that appealing design aesthetics enhance visual attractiveness and lead consumers to perceive greater functional and practical value in a product. Therefore, the following hypothesis is proposed:

**H1:** Design aesthetics positively influences perceived usefulness.

In addition, prior studies have shown that design aesthetics can enhance consumers' perceived authenticity (Wu and Hsu, 2018). Within the context of cultural and creative products, cultural symbols and aesthetic forms contribute to more authentic cultural representation (Zhu and Rahman, 2025). In the fashion context, consumers often rely on factors such as aesthetic style, fashionability, and a sense of heritage when evaluating the authenticity of luxury fashion brands (Lin and Ku, 2023). However, in the context of ethnic cultural products, aesthetic appeal does not necessarily correspond to cultural authenticity. Even when a design is visually attractive, consumers may perceive it as less culturally authentic if it misuses cultural symbols, departs from the original cultural context, or excessively reconstructs traditional cultural elements. Therefore, the positive effect of design aesthetics on perceived authenticity cannot be explained solely by visual attractiveness but should also be understood in terms of the consistency between aesthetic expression and cultural symbols.

Based on this reasoning, this study proposes that when ethnic cultural elements are appropriately represented, high-quality design aesthetics can enhance consumers' perceived authenticity by conveying craftsmanship, cultural consistency, and visual credibility. Therefore, the following hypothesis is proposed:

**H2:** Design aesthetics positively influences perceived authenticity.

#### 2.2.2 Cultural identity

Cultural identity refers to the extent to which individuals recognize and identify with specific cultural elements (Clark, 1990). Such identification involves not only an understanding of cultural characteristics but also the development of a sense of belonging to that culture (Phinney, 1989). In the present study, cultural identity refers to the situational identification that consumers develop with the ethnic cultural symbols and cultural meanings embedded in AI-generated Tujia fashion designs.

Previous studies have shown that cultural identity significantly influences consumers' perceived usefulness (Su et al., 2025). In the context of digital museums, cultural identity has been found to enhance perceived usefulness (Liang and Wang, 2026). Within the fashion domain, digital products based on traditional costumes can foster cultural identity (Fu and Liang, 2022). Similarly, identity-related perceptions have been shown to influence consumers' evaluations of product value (Ma et al., 2021). Based on these findings, this study argues that consumers with stronger cultural identity are more likely to perceive a product as capable of fulfilling their needs for cultural expression, identity construction, and aesthetic identification, thereby enhancing their perceived usefulness. Therefore, the following hypothesis is proposed:

**H3:** Cultural identity positively influences perceived usefulness.

In addition, previous research has demonstrated that cultural identity can enhance consumers' perceived authenticity (Yang et al., 2025). In the context of intangible cultural heritage, cultural identity has been found to exert a significant positive effect on authenticity perceptions, including both constructive authenticity and existential authenticity (Tian et al., 2020). In the fashion context, consumers' identification with Hanfu culture and their cultural motivations have been shown to positively influence authenticity perceptions (Li et al., 2023). Accordingly, this study proposes that when consumers strongly identify with a particular culture, they are more likely to understand the cultural symbols and historical meanings embedded in related products, thereby developing stronger perceptions of authenticity. Therefore, the following hypothesis is proposed:

**H4:** Cultural identity positively influences perceived authenticity.

#### 2.2.3 Perceived usefulness

Perceived usefulness refers to the degree to which an individual believes that using a particular system can enhance performance (Davis et al., 1989). It has also been conceptualized as an individual's subjective perception that a product, system, or technology can satisfy specific needs and help achieve desired goals (Davis, 1989). In the present study, perceived usefulness is defined as the extent to which consumers perceive this type of fashion design as having practical value in terms of everyday wear, fashion coordination, and market acceptance.

The positive effect of perceived usefulness on purchase intention has been well established across studies of consumer behavior, fashion products, and apparel consumption (Hajli, 2014; Harrigan et al., 2021; Hwang et al., 2016; Moslehpour et al., 2018). Therefore, this study treats this relationship as a foundational pathway through which consumers' functional evaluations are translated into purchase responses, rather than as a source of core theoretical novelty. Accordingly, the following hypothesis is proposed:

**H5:** Perceived usefulness positively influences purchase intention.

In addition, prior research has demonstrated that design aesthetics significantly affects perceived usefulness and subsequently influences consumers' purchase-related behaviors (Li and Yeh, 2010). In online shopping contexts, design aesthetics has been found to enhance perceived usefulness, which in turn affects consumers' behavioral intentions (Cyr et al., 2006; Yang et al., 2021). Within the fashion domain, the aesthetic style of smart apparel and perceived usefulness jointly serve as important foundations for consumers' purchase intentions (Shi et al., 2026). Accordingly, this study proposes that design aesthetics can increase purchase intention by enhancing a product's attractiveness and functionality, thereby leading consumers to perceive greater practical value. Therefore, the following hypothesis is proposed:

**H6a:** Perceived usefulness mediates the relationship between design aesthetics and purchase intention.

Related studies have further indicated that cultural identity enhances perceived usefulness and subsequently promotes consumer behavior (Liang and Wang, 2026). In the context of intangible cultural heritage-themed games, cultural identity has been shown to significantly influence consumers' perceptions, while perceived usefulness further increases behavioral intention (Bai et al., 2025). In the fashion context, consumers' perceptions of traditional costume cultural elements influence perceived usefulness, which subsequently affects purchase intention (Fu and Liang, 2022). Based on these findings, this study argues that consumers with stronger cultural identity are more likely to perceive the product as capable of fulfilling their needs for cultural understanding, self-expression, and practical use. This, in turn, enhances perceived usefulness and ultimately strengthens purchase intention. Therefore, the following hypothesis is proposed:

**H6b:** Perceived usefulness mediates the relationship between cultural identity and purchase intention.

#### 2.2.4 Perceived authenticity

Perceived authenticity refers to consumers' subjective judgments regarding whether a product, content, or cultural expression is genuine, credible, and consistent with their existing knowledge and beliefs (Beverland and Farrelly, 2010). It represents an overall evaluation formed on the basis of dimensions such as genuineness, credibility, and cultural consistency (Nunes et al., 2021). In the present study, perceived authenticity refers to consumers' evaluations of whether the ethnic cultural symbols, traditional aesthetic characteristics, and cultural meanings expressed in AI-generated Tujia fashion are genuine and trustworthy, as well as the extent to which these expressions align with their existing cultural understanding.

The positive effect of perceived authenticity on purchase intention has been well established in studies of cultural products, design, and apparel consumption (Li et al., 2023; Pelet et al., 2020; Thomas and Fowler, 2021). Therefore, this study treats this relationship as a foundational pathway through which consumers' authenticity evaluations are translated into purchase responses and proposes the following hypothesis:

**H7:** Perceived authenticity positively influences purchase intention.

In addition, previous research has demonstrated that design aesthetics significantly influences perceived authenticity, which subsequently affects consumers' purchase intentions (Wu and Hsu, 2018). In the context of souvenir consumption, design can enhance purchase intention through increased perceived authenticity (Guo et al., 2024). In the fashion context, apparel that does not conform to consumers' aesthetic expectations may reduce perceived authenticity and consequently weaken purchase intention. Accordingly, this study proposes that design aesthetics not only provides visual appeal but also conveys cultural symbolism and a sense of authenticity, thereby influencing consumers' purchase intentions through perceived authenticity. Therefore, the following hypothesis is proposed:

**H8a:** Perceived authenticity mediates the relationship between design aesthetics and purchase intention.

Related studies have further indicated that cultural identity enhances perceptions of authenticity and subsequently promotes consumers' purchase intentions (Tian et al., 2020). Cultural motivation has been found to positively influence object-based authenticity, while authenticity serves as a mediator between cultural motivation and purchase intention (Oyedele et al., 2025). In the fashion context, consumers' identification with Hanfu culture and their cultural motivations positively influence authenticity perceptions, which in turn affect purchase intention (Li et al., 2023). Based on these findings, this study argues that cultural identity strengthens consumers' judgments of a product's cultural authenticity and further enhances purchase intention through perceived authenticity. Therefore, the following hypothesis is proposed:

**H8b:** Perceived authenticity mediates the relationship between cultural identity and purchase intention.

#### 2.2.5 AI Disclosure

AI disclosure generally refers to explicitly informing individuals that content, products, services, or interaction partners are generated, supported, or provided by artificial intelligence (Baek et al., 2026; Li et al., 2024). Existing studies have operationalized AI disclosure through labels or statements such as “AI-generated,” “AI-designed,” “chatbot identity disclosure,” or “AI disclosure labels” (Luo et al., 2019; Shi and Jiang, 2026). In the present study, AI disclosure refers to whether consumers are explicitly informed that AI was involved in the design process when they are exposed to AI-generated Tujia fashion. Unlike general consumer products, ethnic cultural fashion embodies not only aesthetic and functional attributes but also the historical memory, cultural symbols, and spiritual heritage of a particular cultural group. Consequently, consumers' evaluations of such products depend not only on the final design outcome but also on whether they believe the creator genuinely understands and appropriately conveys the underlying cultural meanings. When a design is explicitly disclosed as AI-generated, consumers may further question whether AI is capable of truly understanding the cultural context and spiritual significance embedded in ethnic cultural symbols, thereby influencing their evaluations of the product's cultural meaning and authenticity.

Previous research has shown that information about an artwork's creator plays an important role in shaping aesthetic evaluations and aesthetic appreciation (Chiarella et al., 2022). Artworks labeled as human-created tend to receive higher ratings in terms of liking, aesthetic appeal, and perceived value (Bellaiche et al., 2023), whereas computer-generated art is often subject to aesthetic bias (Chamberlain et al., 2018). In the fashion context, consumers generally respond more favorably to human-designed apparel than to AI-designed apparel (Lee and Kim, 2024). Based on these findings, this study proposes that AI disclosure, as a source cue regarding the creator, may weaken consumers' positive evaluations of design aesthetics. Therefore, the following hypothesis is proposed:

**H9:** AI disclosure negatively influences design aesthetics.

In addition, previous studies have found that AI-generated content may affect cultural identity when it lacks sufficient cultural context, coherent cultural narratives, or relevant background information (Lai et al., 2025). In the context of artistic creation, AI-generated cultural and creative products based on Yangliuqing New Year paintings have been shown to influence consumers' cultural identity (Zhang B. et al., 2023). Within the fashion domain, cultural identity may be affected by the perceived fashionability of AI-generated apparel (El-Etriby, 2025), and consumers often rely on source information and symbolic cues to determine whether a product conveys authentic cultural meaning (Morhart et al., 2015). Accordingly, this study argues that AI disclosure, as information about the creator, may influence consumers' evaluations of a product's cultural meaning, cultural authenticity, and sense of cultural belonging. Therefore, the following hypothesis is proposed:

**H10:** AI disclosure negatively influences cultural identity.

Related studies have further suggested that AI identity disclosure influences consumers' evaluations of information usefulness (Block, 2026; Chen and Jia, 2026). In the context of generative AI, disclosure of AI usage has been found to affect public judgments regarding research quality, engagement, and perceived usefulness (Chen and Jia, 2026). Based on these findings, this study proposes that AI disclosure may influence perceived usefulness by altering consumers' evaluations of information credibility, functional value, and potential risk. Therefore, the following hypothesis is proposed:

**H11:** AI disclosure negatively influences perceived usefulness.

Furthermore, previous research has shown that AI-designed souvenirs are generally perceived as less authentic than those designed by humans (Kim et al., 2026). In digital marketing contexts, AI disclosure labels have also been found to significantly reduce consumers' perceived authenticity (Shi and Jiang, 2026). In the fashion domain, consumers tend to respond more positively to human-designed apparel than to AI-designed apparel, and this difference is driven by perceived authenticity (Lee and Kim, 2024). For ethnic cultural products, judgments of authenticity also depend on whether cultural symbols are appropriately represented and whether the design reflects an understanding of the underlying cultural context and spiritual significance. Consequently, when the AI-generated origin of a design is explicitly disclosed, consumers may further question whether AI is capable of authentically and appropriately expressing the cultural meanings embodied in ethnic culture, thereby reducing perceived authenticity. Based on this reasoning, this study proposes that AI disclosure, as a cue indicating the creator's identity, may weaken consumers' judgments of a product's or content's authenticity, sincerity, and cultural expression. Therefore, the following hypothesis is proposed:

**H12:** AI disclosure negatively influences perceived authenticity.

#### 2.2.6 Algorithm aversion

Considering that algorithm aversion may influence the process through which consumers translate positive psychological evaluations into purchase intentions, this study incorporates algorithm aversion as a moderating variable. Algorithm aversion refers to individuals' biased evaluations of algorithms, which are reflected in more negative attitudes and behavioral tendencies toward algorithms than toward human decision-makers (Jussupow et al., 2020). It is a multidimensional psychological response that encompasses emotions, attitudes, and cognitive evaluations (Castelo et al., 2019). Its negative characteristics may reduce individuals' willingness to accept AI-related products and services (Mende et al., 2019). In the present study, algorithm aversion refers to consumers' distrust, resistance, and negative psychological tendencies toward AI-generated Tujia fashion. Because this study focuses on consumers' psychological responses to specific AI-generated designs, this construct primarily reflects consumers' algorithm-related attitudes within the focal consumption context, rather than a stable individual characteristic that exists independently of the specific context.

Previous studies have shown that consumers tend to place less trust in and rely less on algorithms (Castelo et al., 2019). Even when algorithms perform well, individuals may still avoid algorithmic recommendations or algorithm-generated outcomes (Dietvorst et al., 2015). Perceived usefulness has been found to significantly influence consumers' purchase intentions, while algorithm aversion plays a moderating role in this relationship (Dietvorst et al., 2015; Luo et al., 2019). Based on these findings, this study argues that algorithm aversion may affect the extent to which consumers translate evaluations of perceived usefulness into purchase intentions. Therefore, the following hypothesis is proposed:

**H13:** Algorithm aversion moderates the relationship between perceived usefulness and purchase intention.

In addition, previous research has identified perceived authenticity as an important factor influencing consumers' purchase intentions toward AI-designed products (Lee and Kim, 2024). At the same time, the theory of algorithm aversion suggests that negative attitudes toward algorithms reduce consumers' acceptance of algorithm-generated outcomes and increase their reliance on credibility and authenticity cues during decision-making processes (Castelo et al., 2019; Jussupow et al., 2020). In the context of AI-generated Tujia fashion, perceived authenticity reflects consumers' judgments regarding whether AI-generated designs are genuine, trustworthy, and consistent with their cultural understanding. Accordingly, this study proposes that algorithm aversion may alter the strength of the relationship between perceived authenticity and purchase intention. Therefore, the following hypothesis is proposed:

**H14:** Algorithm aversion moderates the relationship between perceived authenticity and purchase intention.

## 3 Research methodology

This study employed a questionnaire survey based on scenario-based stimulus materials to investigate the mechanisms through which AI disclosure influences consumers' evaluations of AI-generated Tujia fashion design illustrations and their purchase intentions. Data were collected through the Tencent Questionnaire platform. Partial Least Squares Structural Equation Modeling (PLS-SEM) was applied for data analysis, and SmartPLS was used to assess the reliability and validity of the measurement model, examine the path relationships within the structural model, and test the significance of the mediating and moderating effects. In addition, Importance–Performance Map Analysis (IPMA) was conducted to identify key factors for improving purchase intention, thereby enhancing the practical implications and explanatory value of the research findings.

### 3.1 Stimulus materials and AI disclosure manipulation

This study first developed the stimulus materials for the questionnaire. Using Tujia costume as a representative case of Chinese ethnic-minority fashion, the research team identified representative clothing elements and visual characteristics based on Tujia costumes, Tujia brocade, and related motif materials. These elements were then used to generate Tujia fashion design illustrations through generative AI tools. To ensure consistency across the stimulus materials, image proportions, presentation angles, background styles, and garment display formats were standardized throughout the generation process.

A total of 120 AI-generated fashion design illustrations were initially produced. These designs were first screened by three experts from relevant fields. The selection criteria included: (1) clear images with complete garment presentation; (2) clear representation of Tujia motif characteristics; (3) sufficient variation among design concepts to avoid highly repetitive visual expressions; and (4) coverage of different motif themes. Based on these criteria, the research team organized and categorized the generated images and selected representative designs from each motif category. As a result, 15 candidate design illustrations were chosen for expert evaluation.

To ensure the content validity of the stimulus materials, an expert review was subsequently conducted. The expert panel consisted of seven specialists, including two experts in fashion design, two experts in ethnic costume studies, one expert in intangible cultural heritage research, one expert in art and design, and one expert in cultural and creative studies. The candidate designs were evaluated on four dimensions: (1) ethnic cultural expression; (2) AI generation quality; (3) appropriateness of motif application; and (4) aesthetic performance. Each expert independently rated the designs using a five-point Likert scale, and the research team ranked the illustrations according to the average scores across all dimensions.

The evaluation results indicated that the Yangquehua motif designs received the highest ratings, with the two selected illustrations obtaining mean scores of 4.31 and 4.28, respectively. The Peony motif designs achieved mean scores of 4.13 and 4.03, while the Yizihua motif designs received mean scores of 3.94 and 3.78. Overall, all selected illustrations achieved average scores above 3.70, indicating broad expert agreement regarding their effectiveness in ethnic cultural expression, appropriateness of motif application, and overall aesthetic quality. Based on the expert evaluation results, the six highest-rated design illustrations were ultimately selected as the stimulus materials for the formal questionnaire survey.

AI disclosure was manipulated through source labels. Participants were randomly assigned to either the AI disclosure group or the non-disclosure group, with an equal probability of assignment (50%) to each group. Participants in the AI disclosure group viewed the design illustrations accompanied by the label “AI-generated,” whereas those in the non-disclosure group viewed the same illustrations without any information regarding their AI-generated origin. Specifically, the “AI-generated” label was displayed in bold gray text and positioned in the blank background area at the middle right side of each illustration. The label was separated from the garment itself and did not obscure the garment silhouette, patterns, or any other key visual elements. The non-disclosure condition used the identical design illustrations, image size, background, garment presentation angle, and page layout but did not display any source label. Therefore, the only intentional difference between the two experimental conditions was the presence or absence of the “AI-generated” source information. To further illustrate the presentation format and visual salience of the AI disclosure manipulation, examples of the two experimental conditions are shown in Figure 2.

**Figure 2:**
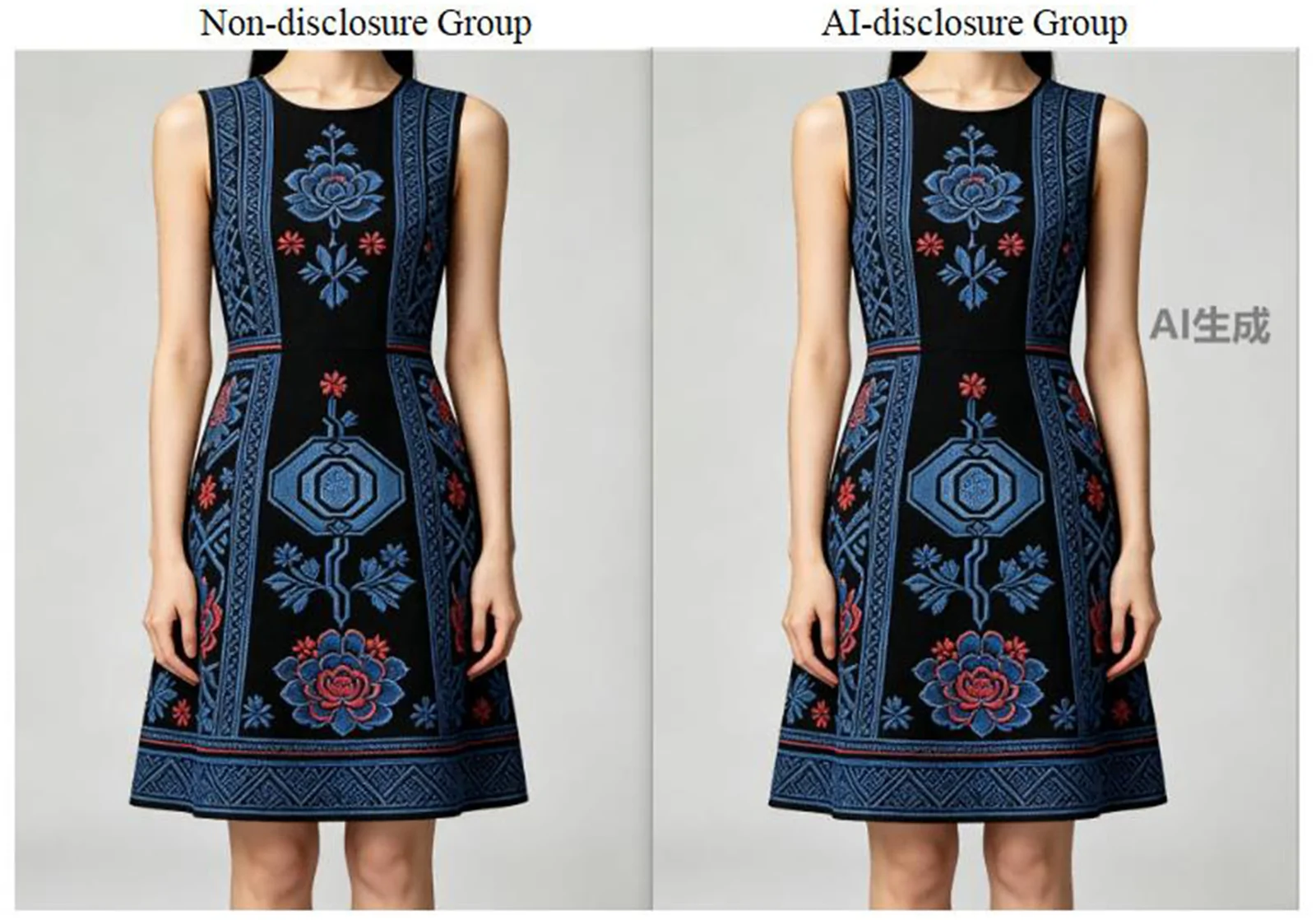
Examples of the AI disclosure manipulation. The label in the right panel was translated as “AI-generated.”

Participants in both groups viewed the same fashion design illustrations, with the only difference being whether information about the AI-generated origin of the designs was disclosed. Accordingly, AI disclosure was operationalized as a binary variable in this study, with the non-disclosure group coded as 0 and the AI disclosure group coded as 1. It should be noted that the expert review was conducted solely to ensure the content validity of the stimulus materials in terms of ethnic cultural expression, appropriateness of motif application, AI generation quality, and aesthetic performance. In contrast, the AI disclosure manipulation was implemented through source labels displayed on the questionnaire pages. After participants completed the purchase intention measures, all participants were informed that the designs had been generated using AI, after which the algorithm aversion measures were administered. Because algorithm aversion was measured after participants had been informed that the designs were AI-generated, it primarily reflects consumers' context-specific attitudes toward AI involvement in the design process rather than a stable individual characteristic independent of the specific consumption context.

### 3.2 Sample and data collection

Participants were recruited from the general consumer population without restricting eligibility to any specific ethnic group, geographic region, or occupational group. Prior to formal data collection, ethical approval was obtained from the Ethics Committee of Fujian University of Technology (Approval No. FJUT-J2026067). All research procedures adhered to the principles of voluntary participation, informed consent, anonymity, and confidentiality. An informed consent statement was presented on the first page of the questionnaire, clearly explaining the research purpose, response requirements, data usage, anonymity protection, and participants' right to withdraw. Participants were required to provide informed consent before proceeding to the formal survey.

A pilot study involving 68 participants was conducted prior to the formal survey to evaluate the clarity of questionnaire items, participants' understanding of the stimulus materials, and the overall survey procedure. Appropriate revisions were made based on the feedback received. The pilot-test results showed a Kaiser–Meyer–Olkin (KMO) value of 0.884, and Bartlett's test of sphericity was significant (*p* < 0.001), indicating that the data were suitable for factor analysis (Hair et al., 2021). Cronbach's alpha values ranged from 0.861 to 0.968, while composite reliability (CR) values ranged from 0.910 to 0.979, all exceeding the recommended threshold of 0.70 (Nunnally, 1975). In addition, the average variance extracted (AVE) values ranged from 0.772 to 0.940, all exceeding the recommended threshold of 0.50, indicating satisfactory internal consistency reliability and convergent validity (Fornell and Larcker, 1981; Liu and Sun, 2025). Therefore, the pilot-test results supported the use of the questionnaire in the formal survey.

The formal survey was administered through the Tencent Questionnaire platform. Participants were recruited using convenience sampling, and the questionnaire link was distributed through social networking channels. Participants were required to be able to read and understand the questionnaire and to participate voluntarily. To ensure that participants were adequately exposed to the stimulus materials, a mandatory confirmation item stating “I have viewed all materials” was presented before respondents could proceed to the questionnaire. This item was used only to confirm that participants had viewed the stimulus materials and did not constitute an independent manipulation check of the “AI-generated” disclosure label.

A total of 450 questionnaires were collected. After excluding responses with excessively short completion times, obvious response patterns, or other indicators of invalid responses, 410 valid questionnaires were retained, yielding a valid-response retention rate of 91.11%. The AI disclosure group and the non-disclosure group each consisted of 205 participants (50%). According to the commonly accepted sample size guidelines for PLS-SEM, the final sample size was sufficient for model estimation and statistical testing (Hair et al., 2019).

### 3.3 Measurement of variables

Except for AI disclosure, which was manipulated as a binary experimental variable, all other constructs were measured using established scales adapted to the context of AI-generated modern fashion design illustrations incorporating Tujia cultural elements. All measurement items employed a seven-point Likert scale, ranging from 1 (“strongly disagree”) to 7 (“strongly agree”).

Design aesthetics was used to assess consumers' evaluations of the visual attractiveness, overall harmony, and aesthetic quality of the design illustrations. Cultural identity measured the extent to which consumers identified with the cultural symbols and cultural meanings of Tujia culture. Perceived usefulness assessed the extent to which consumers believed that the design illustrations possessed practical value in terms of everyday wear, fashion coordination, and market acceptance. Perceived authenticity measured the extent to which consumers perceived the designs as genuine, credible, and appropriate representations of Tujia cultural meanings. Algorithm aversion assessed consumers' distrust of and resistance toward AI-generated designs. Purchase intention measured consumers' willingness to purchase or consider purchasing similar products in the future.

All measurement items were adapted from established studies and modified to fit the context of the present research. The specific measurement items and their sources are presented in Table 1.

**Table 1:** Measurement items and sources.

Construct	Content	References
Design Aesthetics	These fashion designs are aesthetically pleasing.	Li and Yeh (2010); Zhang and Wang (2023)
	The overall visual appearance of these fashion designs is attractive.	
	These fashion designs convey a contemporary sense of fashion.	
Cultural identity	These fashion designs allow me to appreciate the unique charm of ethnic culture.	Xu et al. (2022); Zhang M. et al. (2023)
	These fashion designs create a sense of cultural closeness for me.	
Perceived usefulness	I believe these fashion designs are suitable for everyday wear	Hwang et al. (2016); Liang et al. (2020)
	I believe these fashion designs can meet the needs of contemporary fashion styling.	
	I believe this type of fashion design has the potential to be accepted by the general public.	
Perceived authenticity	Overall, I believe these fashion designs effectively reflect the characteristics of traditional ethnic costumes	Li et al. (2023); Ramkissoon and Uysal (2011)
	I believe these fashion designs preserve the distinctive features of traditional motifs.	
	I believe these fashion designs reflect an authentic ethnic cultural style.	
Purchase intention	If given the opportunity, I would be willing to purchase similar apparel products (for myself or for others).	Lin et al. (2024); Shi et al. (2026)
	If the price and quality were appropriate, I would consider purchasing this type of apparel product (for myself or for others).	
	If similar products become available in the future, I would include them among my purchase options (for myself or for others).	
Algorithm aversion	I feel some resistance toward the involvement of AI in ethnic fashion design.	Shen et al. (2026); Wu et al. (2024)
	The fact that these fashion designs are AI-generated reduces my favorable impression of them.	
	Compared with AI-generated designs, I prefer apparel designed by human designers.	

## 4 Results

### 4.1 Descriptive statistical analysis

The demographic characteristics of the sample are presented in Table 2. A total of 410 valid responses were retained for the final analysis, with 205 participants assigned to the AI disclosure group and 205 participants assigned to the non-disclosure group. Among the respondents, 168 were male (41.0%) and 242 were female (59.0%). Regarding age distribution, the sample was predominantly composed of individuals aged 18–24 years, with 334 respondents accounting for 81.5% of the total sample, indicating that the participants were primarily young adults.

**Table 2:** Demographic characteristics and randomization balance between experimental conditions.

Variable	Category	Non-disclosure group (*n*)	AI-disclosure group (*n*)	Total n (%)	χ^2^	*p*
Gender	Male	88	80	168 (41.0%)	0.645	0.422
	Female	117	125	242 (59.0%)		
Age	18–24 years	161	173	334 (81.5%)	3.817	0.431
	25–30 years	33	23	56 (13.7%)		
	31–40 years	9	6	15 (3.7%)		
	41–50 years	0	1	1 (0.2%)		
	≥50	2	2	4 (1.0%)		
Education	High school diploma, vocational school, or below	8	8	16 (3.9%)	0.268	0.874
	Associate's degree or bachelor's degree	177	180	357 (87.1%)		
	Master's/Ph.D. or higher	20	17	37 (9.0%)		
Total		205	205	410 (100%)		

In terms of educational background, respondents holding an associate's or bachelor's degree constituted the largest proportion of the sample, with 357 participants (87.1%). Respondents with a master's degree, doctoral degree, or higher accounted for 37 participants (9.0%), while those with a high school education, vocational school education, or below accounted for 16 participants (3.9%).

To evaluate the effectiveness of the random assignment, demographic characteristics were further compared between the AI disclosure group and the non-disclosure group. Chi-square tests indicated no significant differences between the two groups in terms of gender, age, or educational level (all *p* > 0.05), suggesting satisfactory demographic balance across the two experimental conditions. The detailed results are presented in Table 2.

In addition, all participants completed the mandatory “I have viewed all materials” confirmation item, indicating that they had completed the viewing of the stimulus materials. It should be noted that no separate item was included to assess participants' recognition of the AI-generated source. Therefore, this confirmation item was used solely to verify that participants had viewed the stimulus materials and did not constitute an independent manipulation check of the AI disclosure label.

Overall, the sample was predominantly composed of young adults with a junior college or bachelor's degree. Accordingly, the findings primarily reflect this population's evaluations of AI-generated Tujia fashion designs. Future research should recruit more diverse samples to further examine the generalizability of the findings across different age groups and cultural backgrounds.

### 4.2 Collinearity assessment

To examine whether multicollinearity was present in the structural model, the variance inflation factor (VIF) values of the inner model were assessed. The results showed that the VIF values for all structural paths ranged from 1.000 to 2.078, which were well below the recommended threshold of 3.3 (Kock, 2015). These findings indicate that no serious multicollinearity issues existed in the model, supporting the subsequent assessment of the structural path coefficients.

### 4.3 SEM-based analysis of the purchase intention model for AI-generated Tujia fashion

#### 4.3.1 Measurement model assessment

Before evaluating the structural model, the measurement model was first assessed to examine the reliability and validity of the measurement instrument. As this study employed the PLS-SEM approach for empirical analysis, the reflective measurement model was evaluated in terms of four aspects: indicator reliability, internal consistency reliability, convergent validity, and discriminant validity.

It should be noted that during the preliminary assessment of the measurement model, item CI1 within the cultural identity construct exhibited semantic overlap with items measuring perceived authenticity, resulting in relatively weak discriminant validity between the two constructs. Therefore, CI1 was removed, while CI2 and CI3 were retained as indicators of cultural identity. After the removal of CI1, the cultural identity construct continued to demonstrate satisfactory reliability and convergent validity.

Regarding indicator reliability, the assessment was based on the outer loadings of the observed variables. As shown in Table 3, the factor loadings of all measurement items ranged from 0.789 to 0.969, exceeding the recommended threshold of 0.70. These results indicate that all items adequately reflected their respective latent constructs and exhibited satisfactory indicator reliability (Hair et al., 2019). Although item AA3 within the algorithm aversion construct had a loading of 0.789, which was lower than the other items within the same construct, it still exceeded the recommended threshold of 0.70 and was therefore retained.

**Table 3:** Reliability and convergent validity of the measurement model.

Constructs	Items	Factor loadings	Cronbach's α	CR	AVE
Design aesthetics	DA1	0.9	0.908	0.942	0.844
	DA2	0.924			
	DA3	0.908			
Cultural identity	CI2	0.93	0.864	0.936	0.880
	CI3	0.939			
Perceived usefulness	PU1	0.8	0.891	0.932	0.821
	PU2	0.921			
	PU3	0.907			
Perceived authenticity	PA1	0.9	0.907	0.942	0.844
	PA2	0.899			
	PA3	0.925			
Purchase intention	PI1	0.9	0.966	0.978	0.937
	PI2	0.969			
	PI3	0.968			
Algorithm aversion	AA1	0.9	0.853	0.911	0.775
	AA2	0.920			
	AA3	0.789			

Internal consistency reliability was evaluated using Cronbach's alpha and composite reliability (CR). The results showed that Cronbach's alpha values ranged from 0.853 to 0.966, while CR values ranged from 0.911 to 0.978, all exceeding the recommended benchmark of 0.70. These findings indicate that all constructs demonstrated satisfactory internal consistency reliability (Cheung et al., 2024). Among them, the purchase intention construct exhibited relatively high Cronbach's alpha and CR values, suggesting strong consistency among its measurement items while remaining within an acceptable range.

Convergent validity was assessed using the average variance extracted (AVE). As presented in Table 3, the AVE values of all latent constructs ranged from 0.775 to 0.937, exceeding the recommended threshold of 0.50. This indicates that each construct explained a substantial proportion of the variance in its indicators and that the measurement model demonstrated satisfactory convergent validity (Cheung et al., 2024).

Discriminant validity was first assessed through cross-loadings. As shown in Table 4, each measurement item exhibited a higher loading on its corresponding construct than on any other construct, indicating that the items adequately distinguished among the different latent variables (Hair et al., 2019). Furthermore, the Fornell–Larcker criterion results presented in Table 5 showed that the square root of the AVE for each construct exceeded its correlations with all other constructs, supporting acceptable discriminant validity of the measurement model (Fornell and Larcker, 1981). Although a relatively high correlation was observed between cultural identity and perceived authenticity, the removal of CI1 improved the discriminant validity between these two constructs. Moreover, both the cross-loading analysis and the Fornell–Larcker assessment supported the treatment of cultural identity and perceived authenticity as distinct constructs in the subsequent analyses.

**Table 4:** Cross-loadings of measurement items.

Items	DA	CI	PU	PA	PI	AA
DA1	**0.925**	0.666	0.676	0.619	0.637	0.090
DA2	**0.924**	0.663	0.687	0.590	0.642	0.061
DA3	**0.908**	0.645	0.730	0.570	0.707	0.058
CI2	0.668	**0.937**	0.547	0.774	0.550	0.058
CI3	0.676	**0.939**	0.665	0.711	0.641	0.115
PU1	0.625	0.538	**0.890**	0.422	0.699	0.127
PU2	0.756	0.627	**0.921**	0.541	0.773	0.118
PU3	0.677	0.588	**0.907**	0.533	0.726	0.200
PA1	0.590	0.740	0.534	**0.931**	0.536	0.084
PA2	0.577	0.706	0.486	**0.899**	0.492	0.069
PA3	0.612	0.734	0.502	**0.925**	0.550	0.039
PI1	0.704	0.626	0.786	0.547	**0.968**	0.126
PI2	0.696	0.602	0.784	0.551	**0.969**	0.144
PI3	0.694	0.617	0.781	0.567	**0.968**	0.143
AA1	0.067	0.070	0.147	0.044	0.143	**0.925**
AA2	0.040	0.057	0.148	0.046	0.124	**0.920**
AA3	0.099	0.127	0.136	0.103	0.105	**0.789**

Bold values indicate the loadings of each item on its corresponding construct.

**Table 5:** Discriminant validity.

Items	DA	CI	PU	PA	PI	AA
DA	**0.919**					
CI	0.716	**0.938**				
PU	0.760	0.647	**0.906**			
PA	0.646	0.791	0.553	**0.919**		
PI	0.721	0.636	0.810	0.573	**0.968**	
AA	0.076	0.093	0.163	0.069	0.142	**0.880**

The bold diagonal values represent the square root of AVE, and the lower triangle shows the Pearson correlations.

Overall, the measurement model met the recommended criteria for indicator reliability, internal consistency reliability, convergent validity, and discriminant validity. These results indicate that all latent constructs demonstrated satisfactory measurement quality and were suitable for subsequent structural model evaluation.

#### 4.3.2 Structural model assessment

The structural model was evaluated using the Bootstrapping procedure in SmartPLS with 5,000 resamples and a 95% confidence interval. The path coefficients (β), *t*-values, significance levels (*p*-values), and hypothesis testing results are presented in Table 6.

**Table 6:** Hypothesis testing results of the structural model.

Hypothesis	Path	β	Standard deviation	*T* statistics	*p* values	Supported
H1	DA → PU	0.602	0.052	11.481	0.000	Yes
H2	DA → PA	0.153	0.047	3.236	0.001	Yes
H3	CI → PU	0.198	0.054	3.673	0.000	Yes
H4	CI → PA	0.662	0.046	14.475	0.000	Yes
H5	PU → PI	0.710	0.034	21.178	0.000	Yes
H6a	DA → PU → PI	0.427	0.046	9.307	0.000	Yes
H6b	CI → PU → PI	0.140	0.038	3.679	0.000	Yes
H7	PA → PI	0.173	0.036	4.873	0.000	Yes
H8a	DA → PA → PI	0.027	0.011	2.469	0.014	Yes
H8b	CI → PA → PI	0.115	0.024	4.797	0.000	Yes
H9	AI → DA	−0.391	0.095	4.119	0.000	Yes
H10	AI → CI	−0.426	0.093	4.555	0.000	Yes
H11	AI → PU	−0.168	0.067	2.508	0.012	Yes
H12	AI → PA	−0.182	0.063	2.909	0.004	Yes
H13	AA × PU → PI	−0.019	0.029	0.639	0.523	No
H14	AA × PA → PI	0.094	0.035	2.712	0.007	Yes

The results indicate that design aesthetics has a significant positive effect on perceived usefulness (β = 0.602, *t* = 11.481, *p* < 0.001) and perceived authenticity (β = 0.153, *t* = 3.236, *p* = 0.001). Therefore, H1 and H2 are supported. Cultural identity also exerts significant positive effects on perceived usefulness (β = 0.198, *t* = 3.673, *p* < 0.001) and perceived authenticity (β = 0.662, *t* = 14.475, *p* < 0.001), supporting H3 and H4. Regarding the formation of purchase intention, perceived usefulness has the strongest positive effect on purchase intention (β = 0.710, *t* = 21.178, *p* < 0.001), while perceived authenticity also significantly and positively influences purchase intention (β = 0.173, *t* = 4.873, *p* < 0.001). Thus, H5 and H7 are supported.

The mediation analysis further reveals that design aesthetics significantly influences purchase intention through perceived usefulness (β = 0.427, *t* = 9.307, *p* < 0.001), and cultural identity also significantly influences purchase intention through perceived usefulness (β = 0.140, *t* = 3.679, *p* < 0.001). Therefore, H6a and H6b are supported. In addition, design aesthetics influences purchase intention through perceived authenticity (β = 0.027, *t* = 2.469, *p* = 0.014), and cultural identity influences purchase intention through perceived authenticity (β = 0.115, *t* = 4.797, *p* < 0.001). Consequently, H8a and H8b are also supported. These findings indicate that perceived usefulness and perceived authenticity play important mediating roles in the relationships between design aesthetics, cultural identity, and purchase intention.

AI disclosure exerts significant negative effects on consumers' psychological evaluations. Specifically, AI disclosure significantly reduces design aesthetics (β = −0.391, *t* = 4.119, *p* < 0.001), cultural identity (β = −0.426, *t* = 4.555, *p* < 0.001), perceived usefulness (β = −0.168, *t* = 2.508, *p* = 0.012), and perceived authenticity (β = −0.182, *t* = 2.909, *p* = 0.004). Therefore, H9, H10, H11, and H12 are all supported. Because the AI disclosure variable was coded as 0 = non-disclosure and 1 = disclosure, the negative coefficients indicate that the presence of an AI-generated label lowers consumers' evaluations of the aesthetic quality, cultural value, authenticity, and practical value of AI-generated Tujia fashion.

The moderation analysis shows that algorithm aversion does not significantly moderate the relationship between perceived usefulness and purchase intention (β = −0.019, *t* = 0.639, *p* = 0.523). Therefore, H13 is not supported. In contrast, algorithm aversion significantly moderates the relationship between perceived authenticity and purchase intention (β = 0.094, *t* = 2.712, *p* = 0.007), supporting H14. This finding suggests that the higher the level of algorithm aversion, the stronger the positive effect of perceived authenticity on purchase intention. In other words, consumers with higher levels of algorithm aversion rely more heavily on judgments of cultural authenticity when deciding whether to accept and purchase AI-generated Tujia fashion. Overall, all proposed hypotheses were supported except for H13.

To further examine how AI disclosure influences purchase intention through the psychological mechanisms proposed by the S–O–R model, this study employed the bootstrapping procedure to test the specific indirect effects of AI disclosure. The results are presented in Table 7.

**Table 7:** Specific indirect effects of AI disclosure through S–O–R pathways.

Path	β	SD	*T* statistics	*P* values
AI → DA → PU → PI	−0.167	0.044	3.841	0.000
AI → CI → PU → PI	−0.060	0.022	2.717	0.007
AI → CI → PA → PI	−0.049	0.014	3.389	0.001
AI → DA → PA → PI	−0.010	0.005	1.981	0.048

The results indicate that AI disclosure indirectly influenced consumers' purchase intention through multiple psychological pathways. Specifically, the indirect effect of AI disclosure → design aesthetics → perceived usefulness → purchase intention was significant (β = −0.167, *t* = 3.841, *p* < 0.001). In addition, the indirect effect through cultural identity → perceived usefulness was significant (β = −0.060, *t* = 2.717, *p* = 0.007), as was the indirect effect through cultural identity → perceived authenticity (β = −0.049, *t* = 3.389, *p* = 0.001). The indirect effect through design aesthetics → perceived authenticity also reached statistical significance (β = −0.010, *t* = 1.981, *p* = 0.048).

These findings further demonstrate that the effect of AI disclosure on purchase intention can be explained through consumers' internal psychological evaluation process, supporting the psychological transformation mechanism proposed by the S–O–R model in the context of AI-generated ethnic cultural fashion. Because the present study focuses on how AI disclosure exerts its influence through consumers' internal psychological states, AI disclosure was not modeled as a direct antecedent of purchase intention; instead, the analysis focused on its indirect effects through the organism variables.

The explanatory power of the structural model is presented in Table 8. The model demonstrates strong explanatory power for purchase intention (PI), with an *R*^2^ value of 0.686 and an adjusted *R*^2^ value of 0.682, indicating that the model explains 68.6% of the variance in consumers' purchase intentions.

**Table 8:** Coefficients of determination (*R*^2^).

Construct	*R* ^2^	Adj. *R*^2^
DA	0.038	0.036
CI	0.045	0.043
PU	0.606	0.603
PA	0.647	0.644
PI	0.686	0.682

The model also exhibits satisfactory explanatory power for perceived usefulness (PU) and perceived authenticity (PA), with *R*^2^ values of 0.606 and 0.647 and adjusted *R*^2^ values of 0.603 and 0.644, respectively. These results suggest that the proposed model effectively explains consumers' evaluations of the practical value and authenticity of AI-generated Tujia fashion.

In contrast, the explanatory power for design aesthetics (DA) and cultural identity (CI) is relatively limited, with *R*^2^ values of 0.038 and 0.045, respectively. One possible explanation is that these two constructs are primarily influenced by AI disclosure. In addition, factors such as individual aesthetic preferences, cultural experiences, and familiarity with ethnic costumes may also affect consumers' evaluations of design aesthetics and cultural identity.

Overall, the proposed model demonstrates satisfactory explanatory power for the key outcome variables, providing a solid basis for interpreting the structural relationships and mediating mechanisms examined in this study.

### 4.4 Importance–performance map analysis (IPMA)

To further identify practical priorities for enhancing consumers' purchase intentions, this study conducted an Importance–Performance Map Analysis (IPMA) using SmartPLS. IPMA simultaneously considers the total effects of antecedent variables on the target variable and their performance levels. Importance reflects the overall influence of a variable on the target construct, whereas performance indicates the actual performance level of that variable within the sample (Ringle and Sarstedt, 2016). In general, variables with high importance but relatively low performance should be prioritized for improvement. In this study, purchase intention (PI) was specified as the target construct, and its related antecedent constructs were included in the IPMA, namely AI disclosure, design aesthetics, cultural identity, perceived usefulness, perceived authenticity, and algorithm aversion. The results of the IPMA are presented in Table 9.

**Table 9:** IPMA results with purchase intention as the target construct.

Construct	Importance	Performance
AI	−0.437	50.000
DA	0.454	60.487
CI	0.255	71.329
PU	0.710	57.212
PA	0.173	72.290
AA	−0.001	58.596

The results indicate that perceived usefulness exhibits the highest importance (total effect = 0.710), whereas its performance score is only 57.212. This finding suggests that although perceived usefulness is the most critical determinant of purchase intention, consumers' evaluations of the practical value of AI-generated Tujia fashion—such as its wearability, styling value, and applicability—still have considerable room for improvement. Therefore, enhancing perceived usefulness should be regarded as the primary strategy for increasing purchase intention.

Design aesthetics ranks second in importance (total effect = 0.454), with a performance score of 60.487. This result indicates that visual attractiveness, aesthetic appeal, and a contemporary fashion image are also important factors in strengthening consumers' purchase intentions. Cultural identity demonstrates an importance value of 0.255 and a performance score of 71.329, while perceived authenticity shows an importance value of 0.173 and a performance score of 72.290. The relatively high performance levels of these two constructs suggest that respondents generally evaluated the cultural expression and authenticity of the designs positively. However, their overall influence on purchase intention is weaker than that of perceived usefulness and design aesthetics.

In addition, AI disclosure exhibits a negative importance value (total effect = −0.437), indicating an overall negative effect on purchase intention. Combined with the results of the specific indirect effects analysis, this finding suggests that the negative influence of AI disclosure was primarily transmitted through consumers' psychological evaluation processes, including design aesthetics, cultural identity, perceived usefulness, and perceived authenticity. Because AI disclosure is a binary experimental variable, its performance score of 50.000 should not be interpreted in the same manner as the performance levels of psychological constructs. Instead, attention should be directed to its negative total effect. This finding suggests that, in the market presentation of AI-generated Tujia fashion, careful consideration should be given to how AI involvement in the design process is communicated and how negative consumer associations with AI-generated products can be mitigated. These issues are particularly important for product communication and marketing strategies.

Finally, algorithm aversion exhibits a total effect close to zero (total effect = −0.001) and a performance score of 58.596, indicating that its overall direct influence on purchase intention is relatively weak. Overall, the IPMA results suggest that improving perceived usefulness should be the primary entry point for enhancing consumers' purchase intentions, followed by efforts to strengthen design aesthetics. At the same time, the presentation of information regarding AI involvement should be carefully designed during product communication to minimize potential negative associations with AI-generated products.

## 5 Discussion

### 5.1 The roles of perceived authenticity and perceived usefulness in the consumption of AI-generated Tujia fashion

This study found that design aesthetics and cultural identity, through perceived usefulness and perceived authenticity, jointly constitute the key psychological pathways underlying consumers‘ evaluations of AI-generated Tujia fashion. These findings suggest that consumers' acceptance of AI-generated ethnic cultural fashion depends not only on visual aesthetics but also on their evaluations of cultural meaning, practical value, and authenticity.

More specifically, design aesthetics exerts the strongest influence on perceived usefulness, indicating that visual attractiveness and a contemporary fashion image enhance consumers' evaluations of a product's practical value. When AI-generated Tujia fashion demonstrates high aesthetic quality, consumers are more likely to perceive it as suitable for wearing, styling, and integration into everyday consumption contexts. At the same time, the significant positive effect of design aesthetics on perceived authenticity should not be interpreted as implying that visual attractiveness itself is equivalent to cultural authenticity. In the context of ethnic cultural products, aesthetic expression is more likely to serve as a positive cue for authenticity only when it is consistent with the appropriate use of cultural symbols. Notably, the stimulus materials used in this study underwent expert review prior to the formal experiment to evaluate ethnic cultural expression, the appropriateness of motif application, and aesthetic performance. Therefore, the positive relationship observed in this study should be interpreted within the context of culturally appropriate representation.

Meanwhile, cultural identity exerted the strongest effect on perceived authenticity, indicating that consumers' authenticity evaluations of ethnic cultural products rely heavily on their identification with the cultural symbols and meanings embedded in the designs. For AI-generated Tujia fashion, consumers' identification with ethnic culture constitutes an important psychological foundation for the formation of perceived authenticity.

The positive effects of perceived usefulness and perceived authenticity on purchase intention are consistent with previous studies (Hajli, 2014; Harrigan et al., 2021; Hwang et al., 2016; Li et al., 2023; Nunes et al., 2021). Because these relationships have been extensively validated in previous research, the present study regards them primarily as foundational psychological pathways through which consumers' evaluations are translated into purchase responses, rather than as the core theoretical contribution of this study. Specifically, perceived usefulness reflects consumers' evaluations of the product's practical value for wearing, styling, and consumption, whereas perceived authenticity reflects their evaluations of the authenticity of its ethnic cultural expression. Together, these two constructs provide the psychological foundation for understanding the effects of AI disclosure and algorithm aversion.

### 5.2 The effects of AI disclosure and algorithm aversion on consumer judgments

The present study found that AI disclosure significantly reduced consumers‘ evaluations of design aesthetics, cultural identity, perceived usefulness, and perceived authenticity, consistent with previous findings on AI-related bias and the diminished authenticity associated with AI-generated designs (Chiarella et al., 2022; Shi and Jiang, 2026). These findings suggest that AI disclosure functions not only as information about the technological origin of a product but also influences consumers' evaluations of its aesthetic and cultural value.

Particularly in the context of ethnic cultural fashion, this negative response may extend beyond general AI-related bias and reflect the distinctive characteristics of cultural creation. Ethnic cultural fashion embodies the historical memory, cultural symbols, and spiritual significance of a particular cultural group. Consequently, when a design is explicitly disclosed as AI-generated, consumers may question whether AI is capable of genuinely understanding the cultural context and appropriately conveying the cultural meanings embedded in ethnic culture. Such concerns may weaken their judgments regarding the product's cultural legitimacy and authenticity. This interpretation is also consistent with previous findings showing that AI-designed fashion and AI disclosure labels reduce consumers' perceived authenticity (Lee and Kim, 2024).

These findings also suggest that the dual nature of AI disclosure deserves careful consideration. On the one hand, transparency regarding AI involvement is necessary from the perspectives of consumer rights, cultural ethics, and market transparency. The European Union's Artificial Intelligence Act has introduced transparency requirements for certain AI-generated or AI-manipulated content, emphasizing the need to appropriately disclose the artificial origin of such content (Block, 2026). Although the United States has not yet established a unified federal framework for AI content disclosure, the Federal Trade Commission (FTC) has continued to monitor risks related to misrepresentation, impersonation, and deceptive marketing associated with AI-generated content (Federal Trade Commission, 2024).

Regarding moderation effects, algorithm aversion does not significantly moderate the relationship between perceived usefulness and purchase intention but significantly moderates the relationship between perceived authenticity and purchase intention. One possible explanation for the non-significant moderation effect is that perceived usefulness represents a relatively functional and utilitarian evaluation. When consumers perceive AI-generated Tujia fashion as possessing practical value for wearing, styling, or consumption, such functional judgments may operate relatively independently of their overall attitudes toward algorithms. This interpretation aligns with previous studies suggesting that acceptance or rejection of algorithms is task-dependent. Individuals tend to exhibit weaker resistance toward algorithms when tasks are perceived as analytical, functional, or efficiency-oriented (Castelo et al., 2019).

Notably, the positive influence of perceived authenticity on purchase intention becomes stronger as algorithm aversion increases. This finding suggests that consumers with high levels of algorithm aversion do not necessarily reject AI-generated Tujia fashion altogether. Instead, they rely more heavily on authenticity judgments when deciding whether to accept such products. When these consumers perceive AI-generated designs as genuinely and appropriately representing ethnic culture, authenticity may serve as an important psychological mechanism for reducing resistance toward algorithmic design. This result echoes previous findings indicating that consumers often lack trust in algorithms (Castelo et al., 2019) and tend to prefer human judgment (Dietvorst et al., 2015). In the context of ethnic cultural products, consumers' resistance may therefore reflect not only concerns about AI technology itself but also doubts about whether AI can adequately understand and appropriately convey the cultural meanings embedded in ethnic culture. However, this interpretation remains speculative and should be further examined in future research.

### 5.3 Theoretical implications

This study makes three primary theoretical contributions. First, it extends the S–O–R model to the consumption context of AI-generated Tujia fashion. The findings reveal how AI disclosure, as an external stimulus, influences purchase intention through internal psychological states, including design aesthetics, cultural identity, perceived usefulness, and perceived authenticity. This demonstrates that consumers' acceptance of AI-generated Chinese ethnic-minority fashion is shaped by multiple psychological pathways involving aesthetics, culture, functionality, and authenticity. Because several of these relationships have been well established in previous research, they are treated in the present study primarily as foundational mechanisms underlying the formation and transformation of consumer evaluations rather than as the core theoretical contribution of this research. Accordingly, these foundational pathways were retained to preserve the integrity of the S–O–R psychological mechanism, while the theoretical discussion focuses on the negative effects of AI disclosure and the boundary effect of algorithm aversion. This approach reduces unnecessary repetition of well-established relationships and highlights the novel theoretical mechanisms examined in this study.

Second, this study advances understanding of the mechanisms underlying AI disclosure. Unlike previous studies that primarily examined the direct effects of AI disclosure on attitudes or purchase intentions, the present study demonstrates that AI disclosure simultaneously weakens consumers' aesthetic evaluations, cultural identity, perceptions of practical value, and perceptions of authenticity. These findings suggest that AI disclosure functions as a source cue capable of reshaping overall product evaluations. Therefore, one of the central theoretical contributions of this study is to reveal how AI disclosure reconstructs consumers' multidimensional evaluations of AI-generated ethnic cultural fashion, rather than simply revalidating well-established relationships in consumer research.

Third, this study distinguishes between the roles of perceived usefulness and perceived authenticity. Perceived usefulness emerges as the strongest direct predictor of purchase intention, reflecting the utilitarian logic underlying contemporary ethnic fashion consumption. In contrast, perceived authenticity, although exhibiting a weaker direct effect, plays a critical role in transmitting the influence of cultural identity and among consumers with high levels of algorithm aversion, reflecting the authenticity-based logic of AI-generated Chinese ethnic-minority fashion consumption. These findings also contribute to the literature on algorithm aversion by demonstrating that algorithm aversion does not necessarily suppress purchase intention directly but may instead alter the psychological criteria consumers rely upon when making purchase decisions. Accordingly, the negative effect of AI disclosure and the boundary effect of algorithm aversion on authenticity judgments constitute the primary theoretical contributions of this study beyond the existing literature.

### 5.4 Practical implications

The findings of this study provide several practical implications for AI-assisted ethnic cultural fashion design and the development of cultural and creative products. First, designers should prioritize enhancing the perceived usefulness of AI-generated Chinese ethnic-minority fashion. The IPMA results indicate that perceived usefulness has the highest importance for purchase intention, yet its performance level still has room for improvement. Therefore, contemporary fashion design incorporating ethnic cultural elements should extend beyond motif transfer and visual decoration to include considerations of garment silhouettes, usage scenarios, styling possibilities, and market positioning. Such efforts can help consumers recognize the practical value of these products. This recommendation is consistent with previous apparel consumption research suggesting that functional, expressive, and aesthetic needs jointly influence purchase intentions (Hwang et al., 2016; Liang et al., 2020).

Second, companies and design teams should strive to balance design aesthetics and cultural authenticity. AI-generated designs should ensure that ethnic cultural symbols remain recognizable while being naturally integrated into contemporary fashion. Authenticity is particularly important for consumers with high levels of algorithm aversion. Therefore, product communication strategies may benefit from presenting information regarding motif origins, cultural meanings, design transformation processes, and expert evaluation procedures to enhance consumers' trust in the authenticity of cultural expression. This recommendation is consistent with previous findings showing that authenticity significantly influences consumer evaluations and purchase intentions for cultural products (Li et al., 2023).

Finally, AI disclosure should be managed carefully. The results indicate that simply labeling a product as “AI-generated” may reduce consumers' evaluations of aesthetics, cultural identity, usefulness, and authenticity. Consequently, marketing communications should not focus exclusively on AI technology itself. Instead, they should clearly explain the specific role of AI within the design process while emphasizing the involvement of human designers, ethnic cultural experts, and intangible cultural heritage inheritors in cultural selection and final design decisions. Such communication strategies may help reduce negative associations with AI-generated products and increase consumers' acceptance of their cultural value (Bellaiche et al., 2023; Lee and Kim, 2024).

## 6 Conclusion

### 6.1 Research conclusions and contributions

This study investigated the formation mechanism of consumers' purchase intention toward AI-generated Tujia fashion based on the S–O–R model. The results indicate that design aesthetics and cultural identity, through perceived usefulness and perceived authenticity, constitute the psychological pathways through which consumers' evaluations are translated into purchase intention. More importantly, AI disclosure significantly reduced consumers' evaluations of design aesthetics, cultural identity, perceived usefulness, and perceived authenticity. Although algorithm aversion did not significantly moderate the relationship between perceived usefulness and purchase intention, it significantly strengthened the positive effect of perceived authenticity on purchase intention.

The primary contribution of this study lies in introducing AI disclosure and algorithm aversion into the context of AI-generated ethnic cultural fashion. While retaining the established mechanisms through which consumers evaluate products in terms of aesthetics, cultural meaning, practical value, and authenticity, this study further demonstrates the negative influence of AI disclosure on consumers' multidimensional product evaluations and identifies the boundary effect of algorithm aversion on authenticity judgments. In doing so, this study extends the application of the S–O–R model to the consumption of AI-generated cultural products and provides empirical evidence for understanding how consumers evaluate and accept AI involvement in ethnic cultural creation.

### 6.2 Research limitations

Despite its contributions, this study has several limitations. First, the sample consisted exclusively of Chinese consumers and was primarily composed of respondents aged 18–24 years with associate's or bachelor's degrees. Therefore, the findings mainly reflect the evaluation characteristics of younger consumers, and their generalizability to broader age groups, individuals with different educational backgrounds, consumers with different levels of cultural experience, and apparel markets in other countries requires further examination. In addition, this study did not measure or control participants' prior familiarity with Tujia culture. Because cultural familiarity may simultaneously influence cultural identity and perceived authenticity, its potential confounding effect cannot be completely ruled out. Future research should measure and control individual difference variables such as cultural familiarity, experience with generative AI, and fashion involvement, while recruiting more diverse samples to improve the external validity and generalizability of the findings.

Second, this study employed a combination of questionnaire surveys and scenario-based experiments. Although AI disclosure was manipulated experimentally, the relationships among the remaining psychological constructs were analyzed using cross-sectional data. Accordingly, the present study identifies significant associations and theoretical pathways among the variables; however, caution should be exercised when interpreting the findings in relation to long-term changes in consumer behavior or actual purchase behavior. In addition, algorithm aversion was measured after participants had been informed that the designs were AI-generated. Therefore, this construct may reflect consumers' context-specific attitudes toward AI-generated fashion designs, rather than a stable individual characteristic that exists independently of the specific consumption context. Future research could measure algorithm aversion prior to AI information disclosure or employ more stable individual-difference measures to further examine its role in the acceptance of AI-generated products.

Third, although the AI disclosure group and the non-disclosure group viewed identical design illustrations, image sizes, and questionnaire procedures, and the “AI-generated” label was positioned in the blank background area outside the main garment to minimize direct interference with the visual presentation of the clothing, the original questionnaire did not include an independent item assessing recognition of the AI source or recall of the disclosure label. The mandatory “I have viewed all materials” confirmation item could only verify that participants had viewed the stimulus materials and could not directly confirm whether all participants in the AI disclosure group had noticed and processed the disclosure label. Moreover, the presence of the label itself introduced additional visual information for the disclosure group. Therefore, the interpretation of the AI disclosure effect should be made with caution. Future research could include both a source-recognition item and a neutral-label control condition with the same visual format to distinguish the semantic effect of AI disclosure from the potential influence of label visual salience.

Fourth, this study focused exclusively on Tujia fashion as a representative case of Chinese ethnic-minority fashion. Although this context provides a well-defined cultural and design setting, different ethnic cultural symbols, apparel types, levels of AI generation quality, and forms of AI disclosure may elicit different consumer responses. In addition, participants evaluated six design illustrations, and the questionnaire measured their overall evaluations of these designs rather than their responses to each individual stimulus. Consequently, the findings primarily reflect participants' overall evaluations of the entire set of design stimuli and cannot distinguish differences associated with individual designs. Future research could extend the investigation to a broader range of cultural products and fashion design contexts while evaluating different design stimuli separately to further examine the effects of different cultural contexts and design characteristics.

### 6.3 Future research directions

Future research may extend the present study in several ways. First, future studies may broaden the sample by including consumers with different ages, geographic backgrounds, ethnic identities, and levels of cultural familiarity. Comparisons between Chinese consumers and consumers in other fashion markets could help assess cross-cultural differences in the acceptance of AI-generated Chinese ethnic-minority fashion and further examine the cross-cultural applicability of the proposed model. In addition, future research may expand the context to other ethnic fashion traditions, such as Miao, Tibetan, and Mongolian costumes, in order to explore how different cultural symbols and clothing traditions influence consumer response mechanisms.

Second, future studies may adopt longitudinal designs, real-purchase experiments, willingness-to-pay measures, or eye-tracking methods to examine the process through which consumers move from initial evaluations to actual purchasing behavior. Compared with purchase intention, actual purchase decisions, willingness to pay, and recommendation behaviors may provide more direct indicators of market acceptance of AI-generated Chinese ethnic-minority fashion.

Third, future research may compare the effects of different AI disclosure strategies and design contexts. For example, labels such as “AI-generated,” “AI-assisted design,” “designer–AI co-creation,” and “AI design reviewed by cultural experts” may evoke different levels of trust, authenticity perceptions, and algorithm aversion. Future studies may also extend the proposed model to other ethnic cultural products and creative industries, such as cultural souvenirs, digital fashion, virtual skins, jewelry design, and home décor products, in order to examine its applicability across a broader range of cultural consumption contexts.

## Data Availability

The raw data supporting the conclusions of this article will be made available by the authors, without undue reservation.

## References

[B1] Baek T. H. Kim J. Kim J. H. (2026). Effect of disclosing AI-generated content on prosocial advertising evaluation. Int. J. Advert. 45, 171–192. doi: 10.1080/02650487.2024.2401319

[B2] Bai Y. Wu S. Liu R. Zheng F. (2025). Understanding users' behavior intention of serious games for intangible cultural heritage based on the stimulus-organism-response model. Int. J. Hum.-Comput. Interact. 41, 5137–5149. doi: 10.1080/10447318.2024.2357912

[B3] Bellaiche L. Shahi R. Turpin M. H. Ragnhildstveit A. Sprockett S. Barr N. . (2023). Humans versus AI: whether and why we prefer human-created compared to AI-created artwork. Cognit. Res. Princ. Implic. 8:42. doi: 10.1186/s41235-023-00499-6 37401999 PMC10319694

[B4] Beverland M. B. Farrelly F. J. (2010). The quest for authenticity in consumption: consumers' purposive choice of authentic cues to shape experienced outcomes. J. Consum. Res. 36, 838–856. doi: 10.1086/615047

[B5] Biliakovych L. Mykhailova R. Kostiuchenko O. Derman L. Bryl M. (2025). Imperatives of using artificial intelligence in modern clothing design: systematic review. Fashion Style Popular Cult. doi: 10.1386/fspc_00341_1

[B6] Block M. J. (2026). Transparency in human-AI interaction-an analysis of article 50 (1) AI act. Comput. Law Secur. Rev. 61:106327. doi: 10.1016/j.clsr.2026.106327

[B7] Castelo N. Bos M. W. Lehmann D. R. (2019). Task-dependent algorithm aversion. J. Mark. Res. 56, 809–825. doi: 10.1177/0022243719851788

[B8] Chamberlain R. Mullin C. Scheerlinck B. Wagemans J. (2018). Putting the art in artificial: aesthetic responses to computer-generated art. Psychol. Aesthetics Creativity Arts 12:177. doi: 10.1037/aca0000136

[B9] Chen C. Jia X. (2026). When researchers use AI: public trust, ethical judgments, and the perceived value of academic research. AI Ethics 6:223. doi: 10.1007/s43681-026-01039-w

[B10] Cheung G. W. Cooper-Thomas H. D. Lau R. S. Wang L. C. (2024). Reporting reliability, convergent and discriminant validity with structural equation modeling: a review and best-practice recommendations. Asia Pac. J. Manag. 41, 745–783. doi: 10.1007/s10490-023-09871-y

[B11] Chiarella S. G. Torromino G. Gagliardi D. M. Rossi D. Babiloni F. Cartocci G. (2022). Investigating the negative bias towards artificial intelligence: effects of prior assignment of AI-authorship on the aesthetic appreciation of abstract paintings. Comput. Hum. Behav. 137:107406. doi: 10.1016/j.chb.2022.107406

[B12] Clark T. (1990). International marketing and national character: a review and proposal for an integrative theory. J. Mark. 54, 66–79. doi: 10.1177/002224299005400406

[B13] Cyr D. Head M. Ivanov A. (2006). Design aesthetics leading to m-loyalty in mobile commerce. Inf. Manag. 43, 950–963. doi: 10.1016/j.im.2006.08.009

[B14] Davis F. D. (1989). Perceived usefulness, perceived ease of use, and user acceptance of information technology. MIS Q. 13, 319–340. doi: 10.2307/249008 23455082

[B15] Davis F. D. Bagozzi R. P. Warshaw P. R. (1989). User acceptance of computer technology: a comparison of two theoretical models. Manag. Sci. 35, 982–1003. doi: 10.1287/mnsc.35.8.982

[B16] Dietvorst B. J. Simmons J. P. Massey C. (2015). Algorithm aversion: people erroneously avoid algorithms after seeing them err. J. Exp. Psychol. Gen. 144:114. doi: 10.1037/xge0000033 25401381

[B17] El-Etriby E. M. M. (2025). Creating contemporary women's fashion designs inspired by pharaonic art using artificial intelligence applications. Int. Design J. 15, 545–566. doi: 10.21608/idj.2025.396031

[B18] Federal Trade Commission (2024). FTC Proposes New Protections to Combat AI Impersonation of Individuals. Press release. Washington, DC: Federal Trade Commission.

[B19] Fornell C. Larcker D. F. (1981). Evaluating structural equation models with unobservable variables and measurement error. J. Mark. Res. 18, 39–50. doi: 10.1177/002224378101800104

[B20] Fu Y. Liang H. E. (2022). Sinicized exploration of sustainable digital fashion: Chinese game players' intention to purchase traditional costume skins. Sustainability 14:7877. doi: 10.3390/su14137877

[B21] Guo Y. Cui X. Zhao Y. (2024). Handwritten typeface effect of souvenirs: the influence of human presence, perceived authenticity, product types, and consumption goals. J. Hospitality Tourism Manag. 58, 345–353. doi: 10.1016/j.jhtm.2024.02.009

[B22] Hair J. F. Jr. Hult G. T. M. Ringle C. M. Sarstedt M. Danks N. P. Ray S. (2021). Partial Least Squares Structural Equation Modeling (PLS-SEM) Using R: A Workbook. Cham: Springer. doi: 10.1007/978-3-030-80519-7

[B23] Hair J. F. Risher J. J. Sarstedt M. Ringle C. M. (2019). When to use and how to report the results of PLS-SEM. Eur. Bus. Rev. 31, 2–24. doi: 10.1108/EBR-11-2018-0203

[B24] Hajli M. N. (2014). A study of the impact of social media on consumers. Int. J. Mark. Res. 56, 387–404. doi: 10.2501/IJMR-2014-025

[B25] Harrigan M. Feddema K. Wang S. Harrigan P. Diot E. (2021). How trust leads to online purchase intention founded in perceived usefulness and peer communication. J. Consum. Behav. 20, 1297–1312. doi: 10.1002/cb.1936

[B26] He S. (2021). Research on a segmentation algorithm for the Tujia brocade images based on unsupervised Gaussian mixture clustering. Front. Neurorobot. 15:739077. doi: 10.3389/fnbot.2021.739077 34539373 PMC8446199

[B27] Hwang C. Chung T.-L. Sanders E. A. (2016). Attitudes and purchase intentions for smart clothing: examining US consumers' functional, expressive, and aesthetic needs for solar-powered clothing. Clothing Text. Res. J. 34, 207–222. doi: 10.1177/0887302X16646447

[B28] Jacoby J. (2002). Stimulus-organism-response reconsidered: an evolutionary step in modeling (consumer) behavior. J. Consum. Psychol. 12, 51–57. doi: 10.1207/S15327663JCP1201_05

[B29] Jiang S. Fu Y. (2017). “Fashion style generator,” in Proceedings of the 26th International Joint Conference on Artificial Intelligence (IJCAI-17) (Marina del Rey, CA: International Joint Conferences on Artificial Intelligence), 3721–3727. doi: 10.24963/ijcai.2017/520

[B30] Jussupow E. Benbasat I. Heinzl A. (2020). “Why are we averse towards algorithms? A comprehensive literature review on algorithm aversion,” in Proceedings of the 28th European Conference on Information Systems (ECIS 2020), Virtual Conference (Atlanta, GA: Association for Information Systems), 1–16. Available online at: https://aisel.aisnet.org/ecis2020_rp/168/ (Accessed October 14, 2026).

[B31] Kim H. Shin S. Shin H. H. (2026). Enhancing the authenticity of artificial intelligence (AI)-designed souvenirs. J. Hosp. Tour. Res. 50, 726–741. doi: 10.1177/10963480251385697

[B32] Kock N. (2015). Common method bias in PLS-SEM: a full collinearity assessment approach. Int. J. e-Collaboration (ijec) 11, 1–10. doi: 10.4018/ijec.2015100101

[B33] Kreuzbauer R. Keller J. (2017). The authenticity of cultural products: a psychological perspective. Curr. Direct. Psychol. Sci. 26, 417–421. doi: 10.1177/0963721417702104

[B34] Lai S. Tian Y. Zhang Q. (2025). The impact of AI-generated technologies-driven digital cultural heritage platforms on users' offline cultural participation intentions. npj Heritage Sci. 13:574. doi: 10.1038/s40494-025-02148-1

[B35] Lee G. Kim H.-Y. (2024). Human vs. AI: the battle for authenticity in fashion design and consumer response. J. Retail. Consum. Serv. 77:103690. doi: 10.1016/j.jretconser.2023.103690

[B36] Li F. (2020). The digital transformation of business models in the creative industries: a holistic framework and emerging trends. Technovation 92:102012. doi: 10.1016/j.technovation.2017.12.004

[B37] Li T.-G. Zhang C.-B. Chang Y. Zheng W. (2024). The impact of AI identity disclosure on consumer unethical behavior: a social judgment perspective. J. Retail. Consum. Serv. 76:103606. doi: 10.1016/j.jretconser.2023.103606

[B38] Li X. Niu Y. Xu J. (2023). Factors affecting purchase intention of Hanfu: considering product identification, cultural motivation, and perceived authenticity. Behav. Sci. 13:689. doi: 10.3390/bs13080689 37622829 PMC10451945

[B39] Li Y. Li J. (2022). The influence of design aesthetics on consumers' purchase intention toward cultural and creative products: evidence from the palace museum in China. Front. Psychol. 13:939403. doi: 10.3389/fpsyg.2022.939403 35846671 PMC9284263

[B40] Li Y.-M. Yeh Y.-S. (2010). Increasing trust in mobile commerce through design aesthetics. Comput. Hum. Behav. 26, 673–684. doi: 10.1016/j.chb.2010.01.004

[B41] Liang N. Wang X. (2026). Determinants of digital museum users' continuance intention-an integrated model combining an enhanced TAM3 and UTAUT. Sustainability 18:492. doi: 10.3390/su18010492

[B42] Liang Y. Lee S.-H. Workman J. E. (2020). Implementation of artificial intelligence in fashion: are consumers ready? Cloth. Textiles Res. J. 38, 3–18. doi: 10.1177/0887302X19873437

[B43] Lin F.-L. Ku T.-H. (2023). Effect of digital brand experience on luxury fashion brand authenticity, attachment and loyalty. South Afr. J. Bus. Manag. 54, 1–8. doi: 10.4102/sajbm.v54i1.3583

[B44] Lin R. Li X. Xia F. (2024). The influence of AR virtual clothing design elements on Chinese consumers' purchase intention: novelty, craftsmanship, trendiness, and sociability. Design J. 27, 888–910. doi: 10.1080/14606925.2024.2372173

[B45] Liu J. Sun Y. (2025). How prompt language structures influence perceived camera movement and usage intentions in AI-generated video: an S-O-R framework. IEEE Access. 13, 172080–172095. doi: 10.1109/ACCESS.2025.3613754

[B46] Liu L. Zhao H. (2024). Research on consumers' purchase intention of cultural and creative products-metaphor design based on traditional cultural symbols. Plos One 19:e0301678. doi: 10.1371/journal.pone.0301678 38739577 PMC11090307

[B47] Liu Y. (2003). Engineering aesthetics and aesthetic ergonomics: theoretical foundations and a dual-process research methodology. Ergonomics 46, 1273–1292. doi: 10.1080/00140130310001610829 14612319

[B48] Luo X. Tong S. Fang Z. Qu Z. (2019). Frontiers: machines vs. humans: the impact of artificial intelligence chatbot disclosure on customer purchases. Mark. Sci. 38, 937–947. doi: 10.1287/mksc.2019.1192

[B49] Lyu Y. Shi M. Zhang Y. Lin R. (2024). From image to imagination: exploring the impact of generative AI on cultural translation in jewelry design. Sustainability 16:65. doi: 10.3390/su16010065

[B50] Ma J. Hong J. Yoo B. Yang J. (2021). The effect of religious commitment and global identity on purchase intention of luxury fashion products: a cross-cultural study. J. Bus. Res. 137, 244–254. doi: 10.1016/j.jbusres.2021.08.040

[B51] Mahmud H. Islam A. N. Ahmed S. I. Smolander K. (2022). What influences algorithmic decision-making? A systematic literature review on algorithm aversion. Technol. Forecast. Soc. Change 175:121390. doi: 10.1016/j.techfore.2021.121390

[B52] Mehrabian A. Russell J. A. (1974). The basic emotional impact of environments. Perceptual Motor Skills 38, 283–301. doi: 10.2466/pms.1974.38.1.283 4815507

[B53] Mende M. Scott M. L. Van Doorn J. Grewal D. Shanks I. (2019). Service robots rising: how humanoid robots influence service experiences and elicit compensatory consumer responses. J. Mark. Res. 56, 535–556. doi: 10.1177/0022243718822827

[B54] Morhart F. Malär L. Guèvremont A. Girardin F. Grohmann B. (2015). Brand authenticity: an integrative framework and measurement scale. J. Consum. Psychol. 25, 200–218. doi: 10.1016/j.jcps.2014.11.006

[B55] Moslehpour M. Pham V. K. Wong W.-K. Bilgiçli I. (2018). E-purchase intention of Taiwanese consumers: sustainable mediation of perceived usefulness and perceived ease of use. Sustainability 10:234. doi: 10.3390/su10010234

[B56] Nam C. Cho K. Kim Y. D. (2021). Cross-cultural examination of apparel online purchase intention: SOR paradigm. J. Glob. Fashion Mark. 12, 62–76. doi: 10.1080/20932685.2020.1845766

[B57] Nunes J. C. Ordanini A. Giambastiani G. (2021). The concept of authenticity: what it means to consumers. J. Mark. 85, 1–20. doi: 10.1177/0022242921997081

[B58] Nunnally J. C. (1975). Psychometric theory-25 years ago and now. Educ. Res. 4, 7–21. doi: 10.3102/0013189X004010007

[B59] Oyedele A. Saldivar R. Goenner-Munson E. (2025). Consumers purchase decision for cultural products outside their countries of origin: a craft retailing context. Tourism Hosp. Res. 26, 782–798. doi: 10.1177/14673584241313352

[B60] Pelet J.-É. Durrieu F. Lick E. (2020). Label design of wines sold online: effects of perceived authenticity on purchase intentions. J. Retail. Consum. Serv. 55:102087. doi: 10.1016/j.jretconser.2020.102087

[B61] Perry A. (2016). Consumers' acceptance of smart virtual closets. J. Retail. Consum. Serv. 33, 171–177. doi: 10.1016/j.jretconser.2016.08.018

[B62] Phinney J. S. (1989). Stages of ethnic identity development in minority group adolescents. J. Early Adolesc. 9, 34–49. doi: 10.1177/0272431689091004

[B63] Prashar S. Sai Vijay T. Parsad C. (2017). Effects of online shopping values and website cues on purchase behaviour: a study using S-O-R framework. Vikalpa 42, 1–18. doi: 10.1177/0256090916686681

[B64] Ramkissoon H. Uysal M. S. (2011). The effects of perceived authenticity, information search behaviour, motivation and destination imagery on cultural behavioural intentions of tourists. Curr. Issues Tourism 14, 537–562. doi: 10.1080/13683500.2010.493607

[B65] Ringle C. M. Sarstedt M. (2016). Gain more insight from your PLS-SEM results: the importance-performance map analysis. Ind. Manag. Data Syst. 116, 1865–1886. doi: 10.1108/IMDS-10-2015-0449

[B66] Riou J. Guyon H. Falissard B. (2016). An introduction to the partial least squares approach to structural equation modelling: a method for exploratory psychiatric research. Int. J. Methods Psychiatric Res. 25, 220–231. doi: 10.1002/mpr.1497 26482420 PMC6877219

[B67] Ruiz-Viñals C. Pretel-Jiménez M. Del Olmo Arriaga J. L. Miró Pérez A. (2024). The influence of artificial intelligence on generation Z's online fashion purchase intention. J. Theor. Appl. Electron. Commerce Res. 19, 2813–2827. doi: 10.3390/jtaer19040136

[B68] Shen J. Niu Y. Guo Z. Chai Y. (2026). Algorithm aversion among workers in on-demand platforms: an exploration in the context of ride-hailing. Manag. Syst. Eng. 5:4. doi: 10.1007/s44176-026-00060-x

[B69] Shi M. Guo Q. Li H. Lee K. (2026). Understanding purchase intention for genAI-enabled museum cultural and creative products using a SOR model. Sci. Rep. 16:5858. doi: 10.1038/s41598-026-36224-8 41559397 PMC12894683

[B70] Shi Y. Jiang Z. (2026). Consumer responses to AI disclosure labels: the role of novelty and authenticity. Sage Open 16, 1–18. doi: 10.1177/21582440261417793

[B71] Su J. Wang Y. Li Z. (2025). Investigating user acceptance of mobile cultural applications in China: an expanded technology acceptance model (TAM). Int. J. Hum. Comput. Interact. 41, 2920–2935. doi: 10.1080/10447318.2024.2406614

[B72] Thomas V. L. Fowler K. (2021). Close encounters of the AI kind: use of AI influencers as brand endorsers. J. Advert. 50, 11–25. doi: 10.1080/00913367.2020.1810595

[B73] Tian D. Wang Q. Law R. Zhang M. (2020). Influence of cultural identity on tourists' authenticity perception, tourist satisfaction, and traveler loyalty. Sustainability 12:6344. doi: 10.3390/su12166344

[B74] Toufani S. Stanton J. P. Chikweche T. (2017). The importance of aesthetics on customers' intentions to purchase smartphones. Mark. Intell. Plann. 35, 316–338. doi: 10.1108/MIP-12-2015-0230

[B75] Wu S.-L. Hsu C.-P. (2018). Role of authenticity in massively multiplayer online role playing games (MMORPGs): determinants of virtual item purchase intention. J. Bus. Res. 92, 242–249. doi: 10.1016/j.jbusres.2018.07.035

[B76] Wu W. Huang Y. Qian L. (2024). Social trust and algorithmic equity: the societal perspectives of users' intention to interact with algorithm recommendation systems. Decis. Support Syst. 178:114115. doi: 10.1016/j.dss.2023.114115

[B77] Xu N.-n. Ji X.-z. Guo Y.-z. (2022). A study on the relationship between cultural identity, place attachment, and tourist loyalty. J. Tourism Hosp. Manag. 10, 38–50. doi: 10.17265/2328-2169/2022.01.006

[B78] Yang H. Kim J.-H. Song H. (2025). Influences of menu, restaurant ownership, and location on Chinese ethnic diners' authenticity perceptions. SAGE Open 15, 1–15. doi: 10.1177/21582440251341932

[B79] Yang Y. Tang Y. Zhang Y. Yang R. (2021). Exploring the relationship between visual aesthetics and social commerce through visual information adoption unimodel. Front. Psychol. 12:700180. doi: 10.3389/fpsyg.2021.700180 34552529 PMC8450337

[B80] Yuan N. Somthai P. (2025). Research of aesthetic value for enhancing Tujia ethnic costumes as a cultural symbol in Shizhu county. Asian Creative Architect. Art Design 38:e275015. doi: 10.55003/acaad.2025.275015

[B81] Zhang B. Cheng P. Deng L. Romainoor N. H. Han J. Luo G. . (2023). Can AI-generated art stimulate the sustainability of intangible cultural heritage? A quantitative research on cultural and creative products of New Year Prints generated by AI. Heliyon 9:e20477. doi: 10.1016/j.heliyon.2023.e20477 37810838 PMC10556784

[B82] Zhang M. Guo X. Guo X. Jolibert A. (2023). Consumer purchase intention of intangible cultural heritage products (ICHP): effects of cultural identity, consumer knowledge and manufacture type. Asia Pac. J. Mark. Logist. 35, 726–744. doi: 10.1108/APJML-11-2021-0831

[B83] Zhang X. Wang L. (2023). The influence of design aesthetics on the purchase intention of AI-generated products: taking cultural and creative products as an example. Int. Conf. Hum.-Comput. Interact. 657–669. doi: 10.1007/978-3-031-48038-6_43

[B84] Zhou S. Li T. Yang S. Chen Y. (2022). What drives consumers' purchase intention of online paid knowledge? A stimulus-organism-response perspective. Electron. Commerce Res. Appl. 52:101126. doi: 10.1016/j.elerap.2022.101126

[B85] Zhu Q. Rahman R. (2025). Authenticity in souvenir design integrating cultural features of Dunhuang's mural heritage: a qualitative inquiry. Humanit. Soc. Sci. Commun. 12, 1–11. doi: 10.1057/s41599-025-04710-5

[B86] Zong Z. Liu X. Gao H. (2023). Exploring the mechanism of consumer purchase intention in a traditional culture based on the theory of planned behavior. Front. Psychol. 14:1110191. doi: 10.3389/fpsyg.2023.1110191 36860780 PMC9968741

